# Effect of Ultrasonic Pretreatment on Melon Drying and Computational Fluid Dynamic Modelling of Thermal Profile

**DOI:** 10.17113/ftb.58.04.20.6813

**Published:** 2020-12

**Authors:** João Henrique Fernandes da Silva, José Sabino da Silva Neto, Edilene Souza da Silva, Danilo Emídio de Souza Cavalcanti, Patrícia Moreira Azoubel, Mohand Benachour

**Affiliations:** Federal University of Pernambuco, Department of Chemical Engineering, Av. Prof. Arthur de Sá, s/n, Cidade Universitária, Recife-PE, 50740-521, Brazil

**Keywords:** computational fluid dynamics, melon, ultrasound, drying, heat transfer, mass transfer

## Abstract

**Research background:** Drying is one of the most traditional processes of food preservation. Optimizing the process can result in a competitive product on the market regarding its price and quality. A common method in use as a pretreatment to drying is ultrasound. The goal of this work is to analyze different drying methods with and without applying ultrasound (US) pretreatment, on heat and mass transfer, simulating numerically the temperature profile by computational fluid dynamics (CFD).

**Experimental approach:** The melon slices were pretreated with ultrasound for 10 (US10), 20 (US20) and 30 (US30) min at 25 kHz, and the water loss and solid gain were evaluated. Samples were dried at different temperatures (50, 60 and 70 °C). The effective diffusivity was estimated, and experimental data were modelled using empirical models. The airflow in the dryer and the temperature profile in the melon slice were simulated *via* computational fluid dynamics (CFD).

**Results and conclusions:** Ultrasound pretreatment reduced the drying time from 25% (samples US20 and US30 at 50 °C) to 40% (samples US20 and US30 at 70 °C). The two-term exponential model presented the best fit to the experimental data, and the diffusivity coefficients showed a tendency to increase as the time of exposure of the melon to ultrasonic waves increased. Pretreatment water loss and solid gain behaviour and drying kinetic and diffusion data were used to choose the best experimental conditions to be simulated with CFD. The heat transfer modelling through CFD showed that the temperature distribution along the melon slice was representative. Therefore, the profile obtained *via* CFD satisfactorily describes the drying process.

**Novelty and scientific contribution:** The use of simulation tools in real processes allows the monitoring and improvement of existing technologies, such as food drying processes, that involve complex mechanisms, making it difficult to obtain some data. Application of CFD in the drying processes of fruits and vegetables is still very recent, being a field little explored. There is no record in the literature that uses CFD in the drying of melon.

## INTRODUCTION

 Drying is one of the most traditional processes of food preservation. It is widely used for reducing the water content and, thus the water activity, inhibiting or reducing microbial growth and enzymatic reactions, increasing the shelf life without the aid of additives (*1*, *2*). However, to obtain a product with a competitive price, besides quality, the process needs optimizing, and one of the methods used as a pretreatment to drying is ultrasound (*3*-*11*).

 Sound waves propagate mechanically, *i.e.* they need a medium for their propagation, where they cause oscillations. The sound waves have different properties depending on the type of material in which they spread, *i.e.* "elastic" when they propagate in solids, and "acoustic" when in fluids (*12*). The form of transmission of these waves also varies depending on whether they are elastic or acoustic. The elastic wave is transferred as a transverse wave, which causes variable shear stress, or as a longitudinal one, causing contraction and intermittent dilatation. The acoustic waves offer only one type of transmission, the longitudinal one (*12*, *13*). In a predominantly liquid system, acoustic cavitation is the main consequence caused by ultrasonic waves, which suddenly creates, spreads and collapses microbubbles in the material (*14*). In a solid food with high moisture content, the mass transfer rate (water transport) increases if cavitation occurs in the liquid phase (free water), reducing the drying time (*15*).

 Drying presents some difficulties in understanding the mechanisms related to convective heat and turbulent fluid in the exchange zones. For its optimization, it is not enough only to apply synergistic techniques but also to investigate such mechanisms. Therefore, a better understanding of the physical phenomena of convective drying of foods using predictive tools has become important (*16*). Among the used simulation techniques, the computational fluid dynamics CFD has become interesting over the years (*2*, *16*-*21*), solving the Navier-Stokes equations using the finite volume method. The finite volume method solves conservation equations in the physical space by discretizing its integral form. In this method, the domain is subdivided into a finite number of control volumes from the presumption that the relevant properties are effectively conserved. In each control volume, a centroid, where the values of each interest variable are calculated, is formed and from this, interpolation is used to calculate the values of each studied property. In this way, an algebraic equation is formulated uniquely for each control (*22*).

 Considering drying as an important food preservation method, the present work aims to study, experimentally, different melon drying conditions with and without ultrasound pretreatment. Also, numerical simulation of heat transfer using computational fluid dynamics (CFD) served to show the temperature profile of the melon slice.

## MATERIALS AND METHODS

### Material

 Mature melons of the yellow variety (*Cucumis melo* L.) purchased in the local market (Recife, PE, Brazil) were used. The previously selected, washed and peeled raw material was sliced into rectangles (5.0 cm×3.0 cm×0.5 cm). The initial moisture (*X*_0_) of the melon, determined by the oven method at 105 °C/24 h (*23*), was 88.61%.

### Ultrasound pretreatment

 Melon samples were weighed, placed in 100-mL beakers containing distilled water, and placed in an ultrasonic bath (model USC-2580A; Unique, Indaiatuba, Brazil), without mechanical agitation, at approx. 25 °C. Ultrasound application time was 10, 20 and 30 min. The sample/distilled water mass ratio used was 1:4, and the ultrasound frequency was 25 kHz (154 W), according to the literature (*24*). Tests with samples without the application of ultrasonic pretreatment (W/O US) were performed as a control. After predetermined times, the samples were taken from the distilled water, placed on absorbent paper for 10 s to remove excess water, and weighed. After that, water loss (WL) and solid gain (SG) were calculated using the following equations, respectively:

FTB-58-381-e1.eps

where *m*_W0_ is the initial water content in the product (g), *m*_Wt_ is the water content in the product at time *t* (g), and *m*_0_ is the initial mass of the product (g), and

FTB-58-381-e2.eps

where *m*_S0_ is the initial dry mass (g), *m*_St_ is the dry mass at time *t* (g), and *m*_0_ the initial mass of the product (g).

 The moisture content of the samples without ultrasound treatment (W/O US) was 88.16%. Sample moisture content increased to 90.19% after a 10-minute pretreatment with ultrasound (US10), 90.74% after 20-minute ultrasound (US20), and 90.94% after 30-minute ultrasound (US30).

### Drying kinetics

 Convective drying of melon slices with and without (control treatment) ultrasound pretreatment was performed at 50, 60 and 70 °C, using a stainless-steel fixed bed dryer (tray dryer) with a fixed air velocity of 2.0 m/s. The choice of the temperature range in this study was to avoid very long drying time (temperatures below 50 °C) and high temperatures, which would cause the loss of nutritional components (temperatures above 70 °C).

 Samples were weighed using a semi-analytical balance. The time intervals used for weighing were 15 min during the first hour of drying and 30 min until the equilibrium condition was reached (*10*). The drying kinetics study was performed using dimensionless moisture data. The diffusion model based on Fick’s law was used to estimate the effective diffusivity:

FTB-58-381-e3.eps

where *X* is the moisture content (g of water per g of dry mass), *t* is the time (s), y is the coordinated direction and *D*_eff_ is the effective diffusivity of water (m^2^/s).

 The following equation presents the solution to Eq. 3 proposed by Crank (*25*), considering an infinite flat plate, where the effect of shrinkage is not considered, assuming instantaneous thermal equilibrium and moisture on the surface.

FTB-58-381-e4.eps

where *X*_θ_ is the dimensionless moisture, *X*_0_, *X*_t_ and *X*_e_ are initial, mean at time *t* and moisture content at equilibrium (in g of water per g of dry mass), and *δ* is half of the slab thickness (m).

 The linear dependence of the Arrhenius equation, which is a linear function of the logarithm of the diffusivity and the inverse of the temperature, was tested during the drying process using the following equation:

FTB-58-381-e5.eps

where *E*_a_ is the activation energy (kJ/mol), *A* is a drying constant, *R* is the universal gas constant (kJ/(mol∙K)), and *T* is the absolute drying temperature (K).

### Modelling of drying kinetics and estimation of thermal properties

Three empirical models were used for drying data fit (*26*):

FTB-58-381-e6.eps

FTB-58-381-e7.eps

FTB-58-381-e8.eps

where *a*, *k*, *n* and *w* are the empirical constants in drying models, and *t* is time (min).

The fit of all the models to the experimental data and their parameters was verified with TIBCO Statistica v. 10.0 (*27*). The error (E) in percentage between the values observed and predicted by the empirical models was determined according to the following equation:

FTB-58-381-e9.eps

where *V*_P_ is the expected value, *V*_O_ is the observed value, and *N* the number of points considered in the curve.

 To simulate the temperature profile during the melon drying, the thermal conductivity (*k*_p_/(W/m∙K)) and specific heat capacity (*C*_p_/(J/kg∙K)) were estimated using the equations presented below (*19*). The density (*ρ*), necessary for solving the mass, energy and momentum transport equations, was calculated using the mass-volume relationship.

FTB-58-381-e10.eps

FTB-58-381-e11.eps

### Simulation of the temperature profile by computational fluidodynamics

 The computational domain was solved using the finite volume method, responsible for solving the Navier-Stokes equations based on conservative principles (*28*). To better observe the current lines acting directly on the heat transfer coefficient, which affects the temperature profile, and considering the geometric domain simplicity, the refining mesh was of high relevance, bringing better precision to the results. The mass transfer study was not possible because the melon (solid domain in this study) should be characterized as a porous domain and this could not be performed. The computational cost to create a mesh that reproduces the pores present in the melon would be too high, making it impractical. Thus, only the simulation for energy and momentum was performed.

**Fig. 1:**
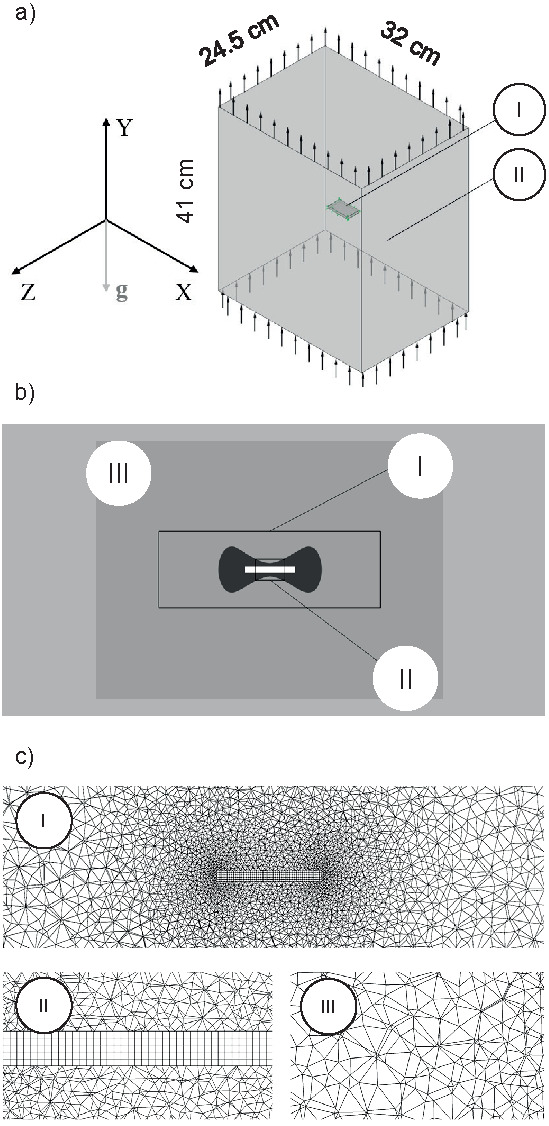
Schematic representation of: a) melon slice (I) and simplified dryer body (II), b) structured and unstructured mesh zones used in the XY plane, and c) denser unstructured zone around the melon (I), structured mesh zone used to represent the melon (II) and less dense mesh area, representing the external fluid medium (drying air) (III)

 The greatest concentration of mesh elements occurred in the wall and contact regions (interface) due to the choice of ‘curvature and proximity’ as criteria to be considered when generating the mesh for the domains. The nodes in the mesh elements were generated by the drop method, which does not generate nodes between the vertices of the geometric element. Due to the importance of the fluid (air) domain, the unstructured mesh was chosen for this domain, which is generated through the Delaunay triangulation and which provides more details for the results. However, the solid domain (melon slice) was represented by a structured mesh, because of its geometric simplicity (*29*, *30*), as shown in Fig. 1.

 Since the trays used here consisted of a network of fine stainless-steel wires, instead of using perforated plates which would affect the airflow into the dryer, they were not considered in the geometric domain used for simulations. Such presumption was necessary since the computational cost associated with the mesh refining would be higher. Also, the wires are very thin, so their interference with the airflow can be neglected.

 Regarding the presumptions made, the effect of shrinkage, generation of heat inside the product, and radiation effects may be neglected. Thermal properties were considered constant. For the turbulence, medium intensity (5%) and eddy (turbulent) viscosity ratio 10, as its use is recommended when there is no information on the turbulence at the entrance, were considered (*30*). The turbulence model used was the shear-stress transport (SST) k–ω (*16*, *19*, *28*), justified due to the high-Re simulation (>10^4^). In the air-dryer and air-slice interfaces, the conservative heat flux was necessary to give stability to the interface model. The selected flow regime was subsonic, due to the low velocities. This work considered transient regime and incompressible fluid, resulting in the following equations:

Conservation of mass (law of continuity):

FTB-58-381-e12.eps

Conservation of momentum (Newton's second law of motion):

FTB-58-381-e13.eps

FTB-58-381-e14.eps

FTB-58-381-e15.eps

Conservation of energy (first principle of thermodynamics):

FTB-58-381-e16.eps

where *T* is the temperature (K), *v*_i_ is the speed (m/s) in direction i and *τ*_ij_ is the stress tensor in the plane i with the flow in direction j, *g* is gravity acceleration (m/s^2^), *ρ* is the melon density (kg/m^3^), p is pressure (Pa), *t* is time (s) and *C*_p_ is specific heat capacity (J/kg∙K).

 The generalized energy equation (Eq. 16) had its velocity terms zeroed, a necessary condition when used for solids.

 The software used to build the geometry, production of the mesh, resolution of the equations, and obtaining the results was the Ansys CFX® 17.0 (*31*). The solutions were considered to have converged at the time when the normalizing residue was less than 10^-4^ (*28*).

## RESULTS AND DISCUSSION

### Drying kinetics

**Fig. 2:**
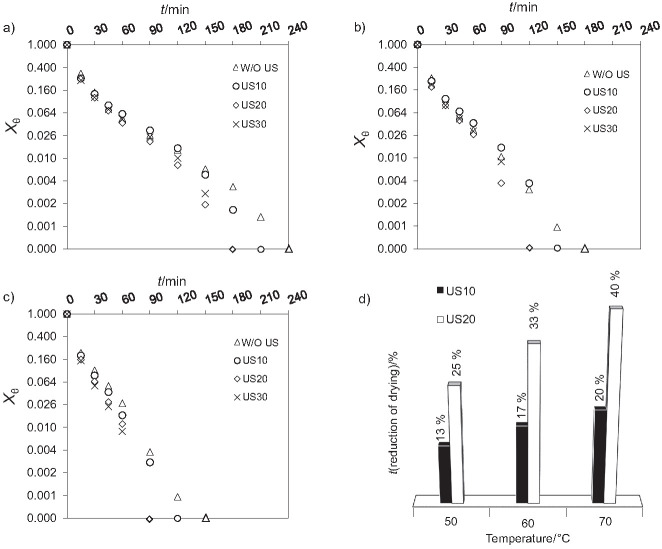
Melon drying kinetics data at: a) 50 °C, b) 60 °C, and c) 70 °C for different treatments, and d) reduction of drying time with the increase of temperature in different treatments. W/O US=drying without ultrasound, US10, US20 and US30=drying with ultrasound pretreatment for 10, 20 and 30 min, respectively. X_ϴ_=is the dimensionless moisture

 The drying kinetics data, obtained at different temperatures, are shown in Fig. 2, where the Y-axis (X_θ_) is in the logarithmic scale. At 50 °C (Fig. 2a), the treatments US20 and US30 required a shorter time to reach the equilibrium condition (180 min), followed by the US10 treatment (210 min). These values represent a reduction in comparison with time obtained in the W/O US treatment (240 min). This reduction is 25% for the US20 and US30 treatments and 12.5% for the US10 treatment. Concerning the kinetics at 60 °C (Fig. 2b), the data presented a similar tendency, where the treatments US20 and US30 also had the shortest time to reach equilibrium (120 min) followed by US10 (150 min) and WUS (180 min). At 70 °C (Fig. 2c), the equilibrium was reached in 90 min (US20 and US30), 120 min (US10) and 150 min (W/O US).

Fig. 2d shows the reductions in drying time in US10 and US20 treatments compared to the W/O US treatment at the different temperatures studied. A synergistic effect was observed between the type of treatment (W/O US, US10, US20 and US30) and the temperature (50, 60 and 70 ° C), so that the reduction in drying time was greater when the temperature and time exposure to ultrasonic waves were also higher. Nowacka *et al.* (*32*) reported similar results, obtaining reductions of 31-40% in the apple drying time at 70 °C, air velocity of 1.5 m/s, frequency of 35 kHz and ultrasound for 10, 20 and 30 min. However, it is also possible to find studies in which the increase in temperature promotes a reduction in drying time (*8*, *33*). This difference, linked to the chaos of the microstructural rearrangement of the product, is best explained below.

### Empirical modelling of experimental kinetics

Table 1 shows the results of the adjustment of the empirical models (Page, two-term exponential, and Henderson and Pabis) to the obtained experimental data. The models gave satisfactory R^2^ values under all the studied conditions, with the two-term exponential and Page models being those that had the best fit. The best suitability of one model over another in the empirical modelling of drying kinetics can be explained by their dependence on operating conditions and characteristics of the material matrix, as observed in different works reported in the literature (*7*, *34*-*37*).

**Table 1:** The coefficient of determination R^2^ for Page (Pg), two-term exponential (TT) and Henderson and Pabis (HP) models for drying kinetics data fittings for different food matrices, model parameters and errors (E) for melon drying

Treatment	Parameter	Temperature/°C								
50			60			70		
TT	HP	Pg	TT	HP	Pg	TT	HP	Pg
W/O US	*a*	0.7353	0.9921	-	0.6638	0.9955	-	0.6086	0.9960	-
	*k*	0.1115	0.0695	0.1813	0.1500	0.0838	0.2178	0.2925	0.0952	0.3211
	*n*	-	-	0.6886	-	-	0.6781	-	-	0.5826
	*w*	0.0272	-	-	0.0395	-	-	0.0444	-	-
	R^2^	0.9999	0.9963	0.9999	0.9999	0.9976	0.9999	0.9999	0.9964	0.9999
	E/%	1.16	35.46	5.21	1.75	41.74	1.81	0.57	38.60	0.63
US10	*a*	0.7377	0.9910	-	0.7223	0.9947	-	0.6253	0.9976	-
	*k*	0.1505	0.0779	0.3212	0.1708	0.0885	0.3257	0.2808	0.1044	0.3297
	*n*	-	-	0.5319	-	-	0.5585	-	-	0.5989
	*w*	0.0242	-	-	0.0317	-	-	0.0498	-	-
	R^2^	0.9999	0.9919	0.9999	0.9999	0.9951	0.9999	0.9999	0.9977	0.9999
	E/%	0.56	43.29	1.16	0.40	46.83	1.79	1.46	40.76	3.96
US20	*a*	0.7367	0.9941	-	0.7092	0.9974	-	0.6335	0.9986	-
	*k*	0.1457	0.0829	0.2746	0.2245	0.1054	0.3840	0.3486	0.1167	0.3586
	*n*	-	-	0.5982	-	-	0.5490	-	-	0.6023
	*w*	0.0297	-	-	0.0409	-	-	0.0572	-	-
	R^2^	0.9999	0.9955	0.9999	0.9999	0.9970	0.9999	0.9999	0.9986	0.9999
	E/%	0.97	43.87	2.43	1.29	51.75	1.20	0.22	43.79	4.02
US30	*a*	0.7529	0.9936	-	0.7676	0.9971	-	0.6800	0.9991	-
	*k*	0.1550	0.0847	0.3309	0.1735	0.1010	0.3555	0.2339	0.1206	0.3333
	*n*	-	-	0.5422	-	-	0.5636	-	-	0.6382
	*w*	0.0266	-	-	0.0345	-	-	0.0589	-	-
	R^2^	0.9999	0.9940	0.9999	0.9999	0.9970	0.9999	0.9999	0.9991	0.9999
	E/%	0.72	45.75	1.43	2.31	58.16	29.34	0.60	43.49	3.14

W/O US=drying without ultrasound, US10, US20 and US30=drying with ultrasound pretreatment for 10, 20 and 30 min, respectively. *a*, *k, n* and *w* are the empirical constants in drying models

**Fig. 3:**
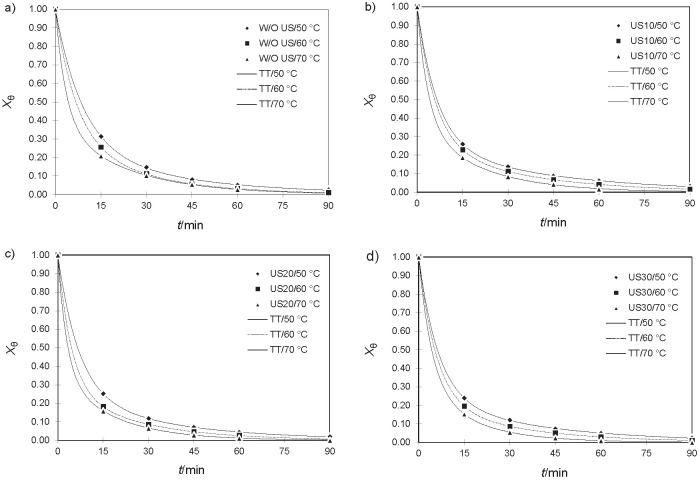
Empirical modelling of experimental data for melon drying: a) without ultrasound (W/O US), and with ultrasound (US) treatment for: b) 10, c) 20 and d) 30 min. The continuous lines represent the best adjusted model, two-term exponential model (TT), for the different temperatures. X_ϴ_ = is the dimensionless moisture

 Despite the high R^2^ obtained in the Henderson and Pabis model, it presented errors varying 35.46-58.16%, which is explained by the distribution of the data along the regression line since the error variance is constant throughout the studied range, that is, the observed responses show homoscedasticity (*38*). Such behaviour was not observed in the Two-term exponential and Page models, which presented low percentage errors. Fig. 3 shows the fitting of the Two-term exponential model, which had the smallest error.

### Mass transfer in terms of effective diffusivity

 The values referring to the variation of the effective diffusivity (*D*_eff_) with the change of temperature, obtained through Eq. 3, are given in Table 2. The increase of the ultrasound exposure time caused an increase in the diffusivity under all conditions. The application of ultrasound facilitates the removal of water, increasing its diffusivity, as emphasized by Zhang *et al.* (*37*), who analyzed the effect of ultrasound on mass transfer and water removal from mushroom slices. However, this effect did not occur when drying US30 samples at 60 °C. The increase in ultrasound time from 20 (US20) to 30 (US30) min at 60 °C reduced the diffusivity by 4.42%. This particular phenomenon, associated with the different temperature effects on the drying time observed in the literature, shows that the microstructural rearrangement of the product is chaotic after exposure to the ultrasonic waves. Thus, the consequences on the effective diffusivity can be unpredictable, depending on the operating temperature range, since the structure can be organized in a way that will benefit or hinder the water transport from the interior to the external part of the product. Other research studies reported similar behaviour. Nowacka *et al.* (*32*), evaluating the effect of ultrasound application on apple drying, found that the application of ultrasound for 10 min resulted in a higher diffusivity coefficient than the treatment for 20 min, generating a difference of 5.07%. Romero and Yépez (*39*), studying the effect of ultrasound as a pretreatment of Andean blackberry (*Rubus glaucus* Benth) to convective drying, noticed that at an air velocity of 3 m/s and 50 °C, the increase of the ultrasound application time from 10 to 30 min caused a reduction of the effective diffusivity of the water of 1.25%. Corrêa *et al.* (*6*), studying the influence of ultrasound application on osmotic pretreatment and subsequent drying of pineapple, also observed a decrease in diffusivity as the time of exposure to the ultrasound increased at 40 °C (6.70%) and 70 °C (2.86%).

**Table 2:** Drying effective diffusivity (*D*_eff_), water loss (WL) and solid gain (SG) after ultrasonic pretreatment of melon slices

Treatment	*D*_eff_ *∙*10^9^/(m^2^/s)						*w*(WL)/%	*w*(SG)/%
323.15 K (50 °C)	R^2^	333.15 K (60 °C)	R^2^	343.15 K (70 °C)	R^2^
W/O US	2.47	0.9965	3.00	0.9969	3.43	0.9974	-	-
US10	2.75	0.9899	3.17	0.9930	3.82	0.9964	-1.19 ±0.17	-1.61 ±0.02
US20	2.97	0.9944	3.85	0.9951	4.33	0.9974	-1.32 ±0.28	-2.20 ±0.03
US30	3.02	0.9914	3.68	0.9953	4.50	0.9983	-2.65 ±0.09	-2.30 ±0.01

W/O US = drying without ultrasound, US10, US20 and US30=drying with ultrasound pretreatment for 10, 20 and 30 min, respectively R^2^=the coefficient of determination

**Fig. 4:**
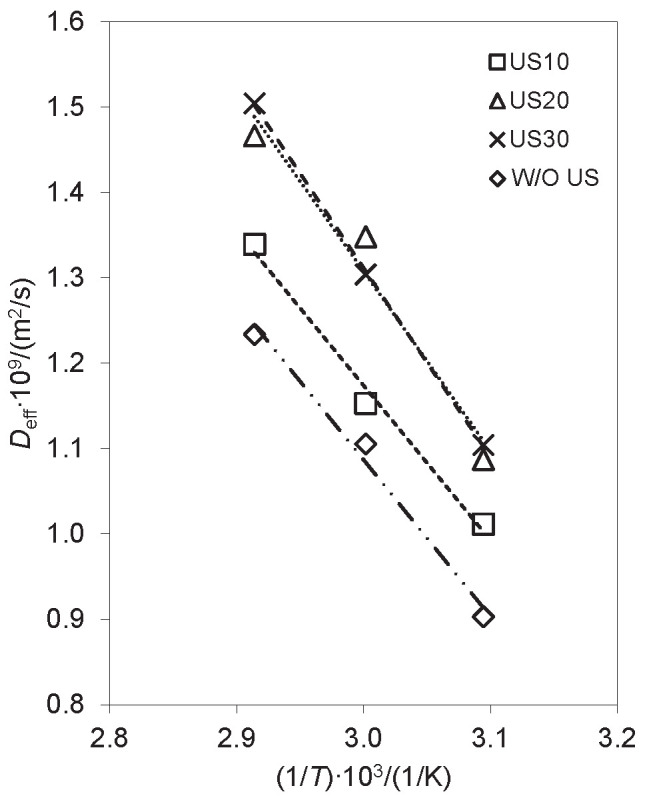
Dependence of the diffusivity on the reciprocal value of temperature W/O US=drying without ultrasound, US10, US20 and US30=drying with ultrasound pretreatment for 10, 20 and 30 min, respectively

Fig. 4 shows the linear profile of the diffusivity with the reciprocal value of temperature, based on Table 2. The data presented a good fit, obtaining R^2^>0.994 for W/O US and US10 treatments, and R^2^>0.977 for treatments US20 and US30. Tzempelikos *et al.* (*19*) found similar R^2^ values, around 0.98.

### Mass transfer in terms of water loss and solid gain

 Two parameters used to determine the conditions for CFD simulation were analyzed: water loss and solid gain, and their obtained values are given in Table 2. The obtained negative water loss values indicated a water gain in all the treatments. As regarding solid gain, the values obtained were also negative, that is, there was a loss of solids. These behaviours corroborate the findings in other studies involving ultrasonic pretreatment for fruit dehydration. Fernandes *et al.* (*24*), for sapota (*Achras sapota* L.) drying, found values of water loss and solid gain ranging from -4.0 to -5.2% and -2.7 to -7.8%, respectively. Garcia-Noguera *et al.* (*40*) obtained values of water loss ranging from -2.7 to -3.9% and of solid gain from -0.1 to -0.7% for strawberry dehydration. Silva *et al.* (*10*), when drying melon slices, obtained values for water loss and solid gain in the range from -8.51 to -10.59% and -0.07 to -1.45%, respectively.

 The treatments US20 and US30 resulted in higher reductions in drying time than W/O US. As these reductions were similar between the two treatments, the use of water loss and solid gain parameters was important for determining adequate conditions. The conditions used for simulation were treatment US20 at 60 °C since water gain (negative water loss) was lower in the US20 treatment than in the US30, and this was the determining factor for the choice, as solid gain values for the two treatments were closer and relied on the dry basis of the product, representing a small fraction of the total mass. The kinetic and diffusion data (Table 2) also corroborated the choice of the US20 treatment at 60 °C as the conditions for simulation.

### Simulation via CFD of the temperature profile of the melon slice

 The values of the properties used in the temperature profile simulation of the melon slice were: *k*_p_=0.5849 (W/O US) and 0.5953 (US20) W/(m∙K), *C*_p_=3891.830 (W/O US) and 3954.830 (US20) J/(kg∙K), *ρ*=2028.467 (W/O US) and 2055.224 (US20) kg/m^3^.

**Fig. 5:**
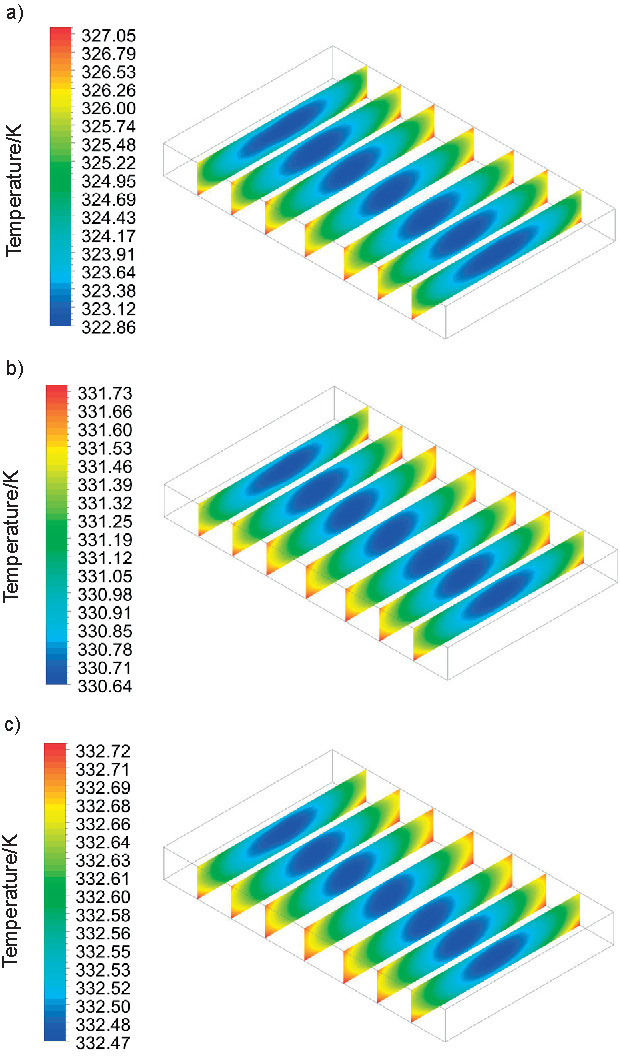
Temperature profile of the melon slice pretreated with ultrasound for 20 min after: a) 30, b) 60 and c) 90 min of drying at 60 °C

Fig. 5 shows the temperature profile of the solid phase of the moist melon over time for US20 treatment at 60 °C. There was also a simulation of W/O US treatment at 60 °C. However, the obtained profile was similar to that of the US20 at 60 ºC (not shown). The geometry was considered unchanged throughout the two processes, and there was only a small difference between the local temperatures when comparing the two treatments since the values ​​of *C*_p_, *k*_P_ and *ρ* were subtly smaller in W/O US at 60 °C.

**Fig. 6:**
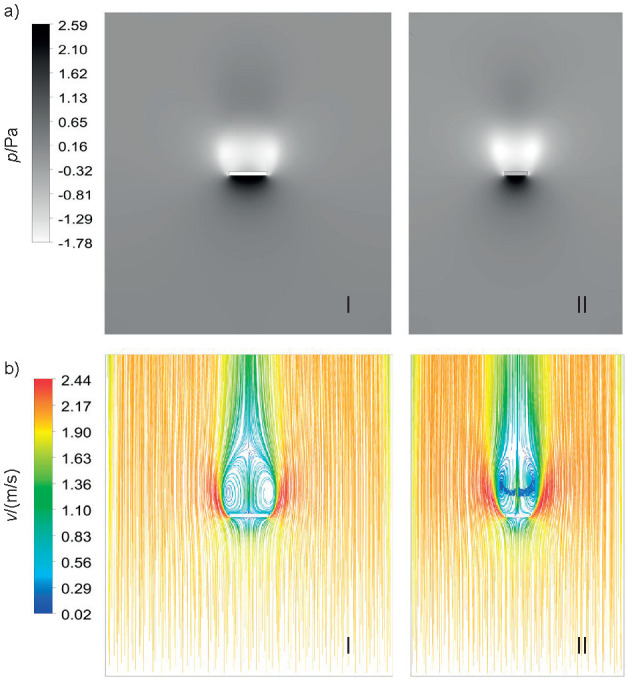
Relative pressure of the: a) YX (I) and YZ (II) planes, and b) current lines in YX (I) and YZ (II) planes

 The results in Fig. 5 show that the temperature is higher at the edges since the proposed geometry generates turbulence in these regions, which in turn increases the local heat transfer coefficient. As it moves away from the lateral zones towards the centre, a decrease in temperature is observed. This occurs through the formation of low-pressure regions above the melon (Fig. 6a), a consequence of the vortexes generated by the lateral air flows (Fig. 6b), reducing the local heat transfer coefficients (*41*). Temperature profiles of fruits and moist objects generated by numerical methods found in the literature are similar to those obtained in this study (*19*-*21*).

## CONCLUSIONS

 The drying kinetics results showed that applying ultrasound as a pretreatment offered a positive synergy with the used temperature. The longer the exposure time to ultrasound, together with the increase in drying temperature, the higher the drying time reduction, reaching up to 40% decrease at 70 °C with the application of ultrasound for 20 min. The empirical model that presented the best fit to the experimental drying data was the Two-term exponential, obtaining R^2^>0.999 and errors of less than 12%. Diffusivity depended on temperature, following the Arrhenius equation. The effective diffusivity coefficient showed a tendency to increase as the melon ultrasound exposure time increased. However, at 60 °C, an anomaly was observed regarding this tendency, since, by increasing the ultrasound time from 20 to 30 min, the effective diffusivity decreased rather than increased. The values of water loss and solid gain were used, together with the kinetic and diffusive data, to choose the best pretreatment condition for computational fluid dynamic (CFD) simulation. The results of the CFD simulation for the temperature distribution along the melon slices were consistent with the data found in the literature. Therefore, the obtained profile satisfactorily describes the drying process.
